# Community needs for the digital divide on the smart city policy

**DOI:** 10.1016/j.heliyon.2023.e18932

**Published:** 2023-08-04

**Authors:** Toddy Aditya, Sinta Ningrum, Heru Nurasa, Ira Irawati

**Affiliations:** a Department Governance Science, Universitas Muhammadiyah Tangerang, Tangerang, Indonesia; b Department Public Administration, Universitas Padjajaran, Bandung, Indonesia

**Keywords:** Digital divide, ICT, Policy implementation, Smart city, Tangerang

## Abstract

This research aims to determine the influence of smart city policy and community needs on the effective implementation of such initiatives, considering the existence of a digital divide within Tangerang City. The digital divide presents a significant challenge for local governments as they strive to ensure equitable and inclusive access to adequate public services for the entire community. A quantitative method of explanatory survey approach was used in this research with questionnaires sent as part of the data collection process in 13 districts of Tangerang City. A total of 400 participants were utilized and data processing was carried out using the SEM-PLS model. Building a smart city was intended to enhance the quality of life by using urban information and technology to increase service effectiveness and cater to community needs. By harnessing information communication technology, the government proactively monitored city dynamics, drove positive changes, and enhanced residents' quality of life. This enabled direct engagement with both residents and infrastructure, making technology an indispensable component of smart city development. Through this mutual utilization, local governments and communities could equally benefit from the advantages and conveniences offered by smart city.

## 1 Introduction

Information communication technology (ICT) presents numerous opportunities for regional development by improving public services [1]. Regions can utilize ICT to streamline service delivery, showcase their potential, and enhance communication with the community and businesses [2]. Tangerang Live application exemplifies one of the potentials of information technology in enhancing public services. This mobile-based application functions as an information hub and public service center, catering to the need of residents.

In line with bureaucratic reforms with the use of ict, Tangerang City as one of the most developed cities in Banten Province, having a geographical location directly adjacent to the DKI Jakarta area, has considerable potential and opportunities for the development of regional competitiveness. This is in line with the vision and mission of the city to realize a livable environment and attract investors and tourism through access to technology [3].

The city of Tangerang which is located west of Jakarta, is one of the fastest growing cities in Indonesia. Significant population growth, economic advancement, and urbanization in the city pose challenges in resource management, public services, and infrastructure development. In facing this challenge, the government has taken steps to increase the utilization of Information and Communication Technology (ICT) and implement smart city initiatives. However, in designing and implementing effective smart city solutions, it is important to understand issues, such as the digital divide and society's need for ICTs. The digital divide refers to differences in access, utilization, and understanding of technology among different groups of people. The diverse needs of society should be the basis for the development of relevant and useful ICT services. To achieve this goal, smart city policy that focuses on digital inclusion, civic participation, and proper data management is needed [4,5].

A new breakthrough made by the Tangerang City government in terms of providing excellent service to the community is through the “Tangerang-Live” application which has advanced to version 4.0. The word “Live” means creating a livable, investor-friendly, visitable, and e-city. The existence of this application aims to realize the vision and mission of Tangerang, namely “the realization of an advanced, independent, dynamic and prosperous environment with a “Moral Karimah” community [6].

Tangerang Live is a single application (Single Sign On) that integrates various existing service applications, resulting in a synergistic technological solution, for easier and more effective functionality. The features provided, include the Live Broadcast service which contains the latest news and information on tourism and tourist destinations, Laksa (suggestion box aspiration service), Emergency 112 enabling the report of urgent events to relevant agencies, licensing services, population services and other economic information) [6]. According to the 2022 ICT report, Tangerang Live application has been downloaded by a total of 253,069 residents.

The development of an e-government, supported by ICT, aims to enhance administrative efficiency, performance, community engagement, and services. It serves as a vital foundation for the advancement of smart government initiatives [7]. Smart city policy is related to the use of application systems in improving public services, and is an important issue in the success of bureaucratic reforms that are on the government's agenda. Smart city development includes 6 (six) main components, namely government, living, mobility, people, environment, and market [8]. The use of technology also plays an important role in enhancing public information disclosure [9], enabling both the government and community to easily monitor and report on the progress of local communities [10].

The realization of smart city should also not only rely on the utilization of information technology, but more importantly in the development of smart people, where individuals who are part of the government system are aware of their participation in realizing smart city [11]. Smart city should be supported by communities, and people who understand their role in development [12]. Potential residents of Tangerang are urged to contribute to finding practical response by creating applications, providing comments on development plans, or carrying out specific jobs or obligations, but they are not urged to contest or supplant core government policy [13] (see Table 1).

Table 2 shows that the users of Tangerang Live application are very small in number, only 12.06% of the total population of the city. Karawaci District is known to consist the most users of the Live application as much as 1.37%. While Benda District has the least users of 0.52%, observed from the large number of residents in each sub-district. This research holds significant importance due to the substantial percentage difference observed. The selection of Tangerang City is because there are still few people who use Tangerang Live application, based on (see Table 2).

**Table 1:** The most users of Tangerang Live Application [14].

No	Description	Scale	Percentage
1	Location	residents of Tangerang City	84%
2	Gender	Man	50,44%
3	Age range	between 30 and 49 years old	52,52%
4	Service	menu Sabakota	95%
5	District	Karawaci	11,36%
6	Types of Work	Private Employees	25,14%.

**Table 2:** Tangerang City Residents and Tangerang live users (2022) [14].

No.	District	Total Population (Thousand)	Live Application Users	Percentage
1.	Cipondoh	203.881	21.312	1,20%
2.	Karawaci	184.216	24.274	1,37%
3.	Pinang	168.477	21.594	1,21%
4.	Tangerang	153.793	20.817	1,17%
5.	Cibodas	149.192	19.890	1,12%
6.	Larangan	143.934	13.876	0,78%
7.	Ciledug	136.525	13.788	0,78%
8.	Periuk	134.741	16.869	0,95%
9.	Neglasari	115.162	15.604	0,88%
10.	Karang Tengah	108.405	12.620	0,71%
11.	Jatiuwung	104.419	12.284	0,69%
12.	Batu Ceper	90.053	11.480	0,65%
13.	Benda	78.294	9.238	0,52%
	**Kota Tangerang**	**1.771.092**	**213.646**	**12,06%**

Several smart city-related research on the utilization of technology in healthcare systems for smart city applications include [15] IoT-based cities [16], Transportation system application of parking system [17], Energy management for Cities Using IoT [18], and energy management in cities [19]. Other research also explained how to minimize disruptions in smart city development in developing countries [20], Stakeholder engagement [21], Measurement techniques and governance policy [22], Natural disaster service platform [23], collaborative governance by Pereira et al. (2018) [24], Urban data platforms, Manage organization and resource management [25], as well as smart city planning and management [26].

Furthermore, research conducted on the alternative conceptions of intelligent citizenship [27,28] using the municipal service technology acceptance model (USTAM) [29], include The effect of smart technology in changing public services, Public trust in the internet is a prerequisite for using e-government services [30], Research [31] in the acceptance and utilization of big data techniques, and [32] Adopting internet banking. Series of research were also conducted [29] In the Challenge of Public Service, ICT creating a digital divide in society [33], ICT for measuring the digital divide by Refs. [34,35] Stating that Generation Z possesses exceptional abilities and demonstrates a superior aptitude in using ict. While [36] many other research highlighted the existence of digital divide among elderly women and mothers.

The research carried out [37] on the implementation of “Live” Tangerang smart city include The effectiveness of public relations communication in the implementation of the “Tangerang Live application” [38], Implementation of e-Government into a smart city (Tangerang Live application case study) [39], The government employs Tangerang Live application as a means to engage and communicate with the local community [40], and Assay of public interest in the use of Tangerang Live application (approach to servqual scale theory) [8].

This research is important because it measures smart city policy and ict-based smart city service (community needs) for the implementation of Tangerang Live application through a digital divide. This is to enhance the knowledge of the implementation and utilization of technology applications in Tangerang, while simultaneously examining the digital divide. The use of digital technology in terms of better governance is a top priority. The use of more intense and innovative web technologies can help public administration modernize the structure and function of government in improving overall performance, from the process of strengthening e-governance to fostering greater transparency, accountability, and engagement between administratives and their citizens.

This research aims to determine the influence of smart city policy and community needs on policy implementation with a digital divide in Tangerang. This is to achieve Tangerang city's vision of becoming a smart city and serve as a pilot model for other regions. As a manifestation of all system development that has been built by Tangerang City regional government, it should also be supported by the community.

## 2 Theoretical background and research hypotheses

### 2.1 Smart city policy

Smart city policy serves as a framework or guide in planning and implementing smart city initiatives. This policy covers strategic decision-making related to ict infrastructure, data use, cybersecurity, community participation, and other aspects related to smart city applications. In theoretical debates, smart city policy is important because it can create a clear foundation, provide the right direction, and regulate the wise use of ICTs. Good policy should also address issues, such as data privacy and security [41,42].

Globalization, with its profound impact on humanity, has caused significant changes, prominently marked by the widespread availability of the internet, an evidence of the remarkable advancement of information technology [43]. Presently, almost all activities carried out by humans intersect with online application, the internet, and social media. Education, religion, sports, business, commerce, politics, government all spread information online and digitally [44]. The use of internet technology gave rise to the policy of e-learning, e-books, e-commerce, e-government, and smart city policy [45].

Smart city development needs to be supported by social, economic, environmental conditions, and with participatory government [46]. Several cities in the world have planned to develop well-established infrastructure and implemented smart city initiatives that are priorities for sustainable development. Acceptance/usage of ict-based smart city service [47] describes several indicators in the acceptance of IT-based smart city technology, including quality of life, innovation policy, personal innovations, city engagement, service quality, perceived privacy, and trust. Smart city policy should be supported by the role of ict infrastructure, although many research also showed the importance of human capital, education, social capital, and environmental interests as drivers of urban growth [48,49].

Smart city according to IBM [50] “is the process of sensing, analyzing, and integrating crucial data from central systems in functioning cities”. Smart city can respond intelligently to a variety of demands, such as necessities of life, environmental protection, public safety, municipal services, business, and industrial operations [51]. It is known to integrate physical, IT, social, and commercial infrastructure to harness the collective intelligence of its residents and resources [52]. A city where ict improves access to public information, services, as well as expression of freedom [53]. In order to de-materialize and expedite bureaucratic procedures and discover innovative solutions to the complexity of city administration, a combination of ict and web 2.0 technologies was merged with other organizational, design, and planning initiatives. This development leads to enhanced sustainability and livability [54]. A smart city serves as a source of inspiration, motivating its citizens to develop and flourish in their personal lives, fostering a culture of sharing information, knowledge, and experiences that enriches life and imparts positively.

Based on expert opinions, a “smart city” can be defined as a technologically driven community that emphasizes the importance of monitoring and integrating services with IT intelligence. This approach aims to optimize access to information and public services while educating citizens about its significance. The term “policy” refers to manner with which the city is structured to work together cohesively as a whole. As a result of the demands of smart city, different ideas evolve in time (or new ones are developed). Large urban areas require majority of the attention while creating smart city, because of their intricate networks and systems. Attention should be given to small city that is rapidly expanding or whose growth is anticipated in the future. The goal is to develop an analysis that can predict the problem, as well as assessing the level of preparedness by providing actionable steps that can be swiftly implemented [55].

The growth of the city is largely dependent on its infrastructure, which is essential to both the operation of commercial organizations and the general well-being of the populace. In urban management, infrastructure is classified into two groups, namely soft (social) and hard (technical). Soft (social), is in the form of social, cultural, and other facilities, while hard (technical) infrastructure, is presented as transportation, telecommunications, water, and energy networks [56]. Smart city based on comprehensive digital systems, builds visual and intelligent scalability urban management and operations. The policy is to use supercomputers and cloud computing to integrate the internet of things by equipping different items with surveillance capabilities. Smart city is the result of harmonizing urban environments with the transformative power of digital technologies, driven by the needs and opportunities presented by the Internet (web technology) [16].

Technologically, its policy aims to build integrated city information and management [57] combining perception, network, and application in achieving a measurable and connected future city according to the needs of the community. Its conceptual framework can be classified into 3 factors, namely technology, people and institutions [58]. This development is able to drive social capital and information technology infrastructure toward a sustainable growth [50]. The policy of a smart city highlights that it is not merely using modern technology, but also a complex ecosystem made up of many stakeholders, including residents, municipal authorities, local and industrial businesses, community, and organizations [59]. It is important to note that the limits of the so-called smart city may extend beyond the actual boundaries, bringing together several governing bodies and municipalities to decide on services on a metropolitan or regional scale [60].

Hypothesis 1 Smart city policy has a positive influence on digital divide

Hypothesis 2 Smart city policy has a positive influence to policy implementation

### 2.2 Community needs for ICT based smart city service

People's need for ICT is becoming an important factor in smart city applications. ICT can improve people's quality of life in various aspects, such as efficiency, comfort, safety, and public participation. Within the framework of smart city, ICT is used to connect city infrastructure, services, and resources to provide better benefits to society. Therefore, understanding the needs of society related to technology and the ability to utilize it is a crucial factor in designing appropriate smart city solutions and applications [61,62].

Building pioneering infrastructure and executing smart city programs are top priorities for municipalities in the globe when formulating economic development strategies [63]. Generally, “smart city” is defined as a dynamic urban environment where comprehensive ICT infrastructure is systematically integrated and implemented, as well as used to enhance social and urban development through increased economic performance, public involvement, and effective governance. Smart city services have not only made more constructive environment, but they have also significantly enhanced the quality of services provided to mobile inhabitants. By leveraging this new layer of technology, cities are capable of delivering superior and more designed services to meet the evolving needs of their mobile population [47]. ICT-enabled smart city services enhance citizens' quality of life, facilitating seamless communication with all government levels and improving their participation in public sector governance [47].

Smart city creates a foundation for human-centered socio-economic well-being and quality of life [64], for instance, ICT applications for the management of intelligent transportation systems, natural resources, energy, water monitoring, buildings, as well as online education and ICT applications for urban health and safety care, electronic service delivery, electronic democracy, and participation in the public sector. To enhance people’ quality of life through ICT-based smart city services, the development of urban management to satisfy their requirements and desires is regarded a successful implementation [47].

To execute innovation and deploy technology, smart city services are a combination of social constructions and technological innovations that interact and influence one another [47]. In particular, it offers a foundation for the growth of highly developed learning and innovation capacities, improving the quality of life for its residents [65].

Services for “smart city” are those that concentrate on modernizing and changing the environment and are often related with urban management of national or municipal governments [66]. When people accept and utilize services after realizing the political execution of smart city services as well as the technical and social values that impact them, the marketing elements that influence their behavior toward acceptance and usage help shape citizen views [67].

To increase municipal infrastructure integration, efficiency, quality of life, transparency, and public involvement, smart city services heavily rely on information [68]. Research showed that consumers might consider technologies, such as e-government and smart cards as a danger, because of the potential for unlawful reuse and unsafe disclosure of their personal information. This is particularly true when it comes to public management of citizen data [69,70]. Presently, delivering public services has caused a rise in the number of privacy-related issues [70].

Hypothesis 3 Community needs have a positive influence on digital divide

Hypothesis 4 Community needs have a positive influence on policy implementation

### 2.3 The digital divide

The digital divide refers to the division or gap in access, utilization, and understanding of ICT among individuals, groups, or regions. In the context of smart city applications, the digital divide is important because it can exacerbate social and economic inequality. To achieve inclusive smart city, efforts are needed to reduce the digital divide through equitable access to ICT infrastructure, digital skills training, and equal opportunities in technology utilization [71,72].

The difference in access to ICT and the usage of the internet for different purposes across people, homes, enterprises, and geographical areas at different socioeconomic levels is known as “digital sniffing” [73]. Various inequalities between nations are reflected in the digital divide. The OECD's (Organization for Economic Co-Operation and Development) member countries' and non-member nations' levels of Internet accessibility for people and enterprises differ significantly. Since it is more accessible and predates the internet and its usage, access to and use of fundamental telecommunications infrastructure is necessary for any discussion of this topic [74].

The OECD countries have started making positive efforts that will guarantee their citizens, businesses, and territories access to these technologies and services. This is because access to and development of information, communication, and e-commerce resources are increasingly seen as being crucial for economic and social development (for efficiency and due to network effects) [75]. To address the digital divide efficiently and effectively, it is crucial for governments to have knowledge on the nature and scope of the gap [76].

The term “digital divide” refers to the disparity of access to ICT, telematics, and the use of the internet for a variety of purposes between individuals, households, businesses, (or communities), and geographic areas at different socioeconomic levels [77]. The digital divide represents the disparities in telematics use and the effects on a nation and across countries [78].

A disparity in access to computers was the first meaning of the phrase “digital divide”. Before the internet was invented, social and economic disparities caused by the development of ICT also resulted in the emergence of a digital gap [34]. It will be challenging for those in poor socioeconomic categories and with low levels of education to access information [33]. Geographical conditions are another consideration, for those who reside in remote and hilly places will find it challenging to acquire information. Due to geographic, economic, and social issues, the digital divide was still an issue during the inception of internet and expanded quickly across society. However, the phrase “digital gap” was changed to include both access to computers and internet [35].

The fundamental factor relating to the disparity between those who have access to digital media and the internet, and those who do not have this opportunity is often referred to as the “digital divide” [79]. The issue of access to computers and the internet has been identified as the digital divide [80]. The digital gap is mostly focused on access disparity, according to Ref. [81]. In the context of the digital divide, the term 'access' initially referred to an individual's ability to connect to the internet. The term “usage” is then used to refer to the availability of opportunities and scheduling options [36].

The unequal development of communication network infrastructure or laws in each area is the root of Indonesia's digital divide [78]. People with good computer and internet access in urban areas will certainly work faster than those who work without computer and internet access [82]. Lack of skills, motivation, and resistance to adopting technology are the causes of the digital divide. Similarly, foreign language skills play a crucial role in internet usage as accessing content can pose challenges in comprehension and understanding [83].

The digital divide encompasses infrastructure access [84], intertwined with ideological, political, economic, social, cultural, geographic, and demographic divisions [85]. The issue of inequality will persist as communication technology advances, and the digital gap serving as a barrier to growth, particularly for poorer nations. Although the proportion is lower than in underdeveloped nations, developed industrialized countries have not yet been able to address the issue of technological inequality, since they continue to experience disparity in a various spheres of life [86].

Hypothesis 5 The digital divide has a positive impact on policy implementation

### 2.4 Implementation analysis framework

A framework for implementation analysis model developed by Ref. [87]. In mapping, this model is labeled “MS” which lies in the “peak down” quadrant and is more on the “forced mechanism” than the “market mechanism”. Mazmanian Sabatier classifies the policy implementation process into three variables.

The challenges related to theoretical and technical implementation, as well as the diverse range of items and intended modifications, should be considered as separate variables when assessing the difficulties and regulatory measures involved. Intervening variables include the ability of the policy to structure the implementation process with indicators of clarity and consistency of objectives, the use of causal theory, the accuracy of source allocation, hierarchical integration among implementing agencies, rules and implementing agencies, the recruitment of implementing officials, and openness to outside parties. Other factors are in terms of measures of socioeconomic, technical circumstances, public support, constituent attitudes, as well as variables outside of policies that have an impact on the implementation process, support of higher officials, commitment, quality of leadership, and implementing officers.

Dependent variables, such as the five stages of the implementation process, including understanding the implementing institution/agency, object compliance, tangible findings, acceptance of results, and finally leading to revisions of the policies implemented.

According to Ref. [87], finding the elements influencing the accomplishment of legal objectives in this process is one of the key roles of implementation analysis. Most research have examined policy implementation, specifically in social programs, such as job training [88], education [89], and health [90]. These studies primarily focused on the interactions between different bureaucracies. In this case, the government officials often defend their established projects or responding to local opposition by resorting to tactics, such as delaying implementation resisting change, and subtly distorting program objectives. A lot of these issues exist because the law was unable to reduce the number of veto/permit points involved and to entrust the implementation to organizations that would give the new program high priority. Additionally, the bureaucracy's decision-making process is complicated and difficult for observer to keep track of it and successfully make their decision.

**Fig. 1:**
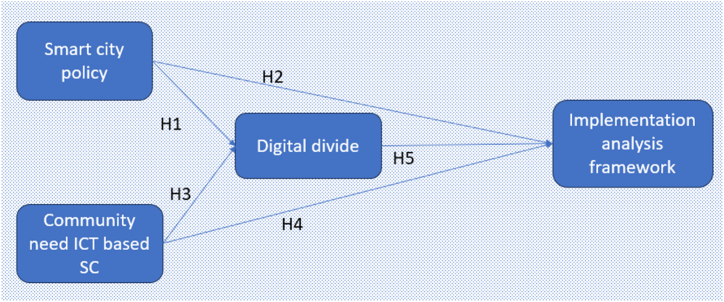
Research model.

In the context of the present technological advancements and urban transformations, model development in Fig. 1, along with the society's demand for ICT and the digital divide, emerge as compelling and pertinent subjects. The concept and definition of smart city as the main framework in research were adopted from Refs. [58,91], and the key components of smart city employed were digital infrastructure, e-governance, ICT availability, integrated public services, and community participation in decision-making [48,72]. According to literature review by Refs. [41,92] people's need for ICT is important because digital technology has changed the pattern of living and interacting within cities. A comprehensive understanding of ICT-related communities' needs, enables us to design solutions that are appropriate and relevant to their requirements. This involves aspects such as internet access, digital public services, public participation in decision making, and the use of technology to improve efficiency and convenience in daily life. The digital divide is important because it affects inclusivity and equality in society [74]. In the digital space, ICT access and skills are key to taking advantage of the opportunities and resources offered by smart city. Understanding the digital divide helps us identify groups of people who are vulnerable to technology inequality, as well as formulate the strategies and policies needed to reduce this gap and ensure that the benefits of smart city development can be extended to all citizens [93,94].

## 3 Method

### 3.1 Research methods

This quantitative research was conducted using an explanatory survey approach. The data collection was conducted through the survey method using a questionnaire filled out by respondents. Direct surveys were conducted by direct observation and giving questionnaires to respondents representing community groups. Online surveys can also be used to cover more geographically dispersed respondents. Data collection was carried out for 2 months (March to April 2022). Leveraging previous literature research and expert judgments, a robust approach was employed to develop a research instrument comprising 71 carefully crafted statements. The data analysis technique used was SEM-PLS (Structural Equation Modeling-Partial Least Square) [95].

This research adheres to a set of ethical principles for research, upholding scientific integrity, human rights, and dignity, which correlate with the guidelines and regulations of Research Ethics Commission (KEP) Universitas Padjajaran no. 17023190042. Ethical approval was obtained from the head of the department of public administration at the faculty of social and political science, Universitas Padjajaran, Bandung. In accordance with the guidelines of the general code of ethics of the Universitas Padjajaran Bandung, active informed consent was obtained from all community who participated in this research.

### 3.2 Units of analysis, population, and sample

This research was conducted in Tangerang municipality due to its strategic position, directly adjacent to DKI Jakarta, being a concentrated resident, trade, and industrial area. The infrastructure in Tangerang is expected to serve as a model that can be replicated and duplicated by other regencies and cities in Indonesia, coupled with the main support in the form of the internet of things (IoT), big data, and cloud computing. This is to ensure that public services and community empowerment in the context of implementing regional development are more optimal. In the future, Tangerang is also expected to be a pilot project in the development of place branding for regency and city areas in Banten Province, as a center of rapid growth to attract domestic and foreign tourists, as well as investors through Soekarno-Hatta International Airport.

The analysis involved all Tangerang City communities who are in 13 districts and have used the Smart City Tangerang Live application as well as those who already carrying out activities [96]. A sampling technique known as cluster sampling was used which involves grouping subjects based on traits such as occupation, gender, or social group. However, it is important to note that this method does not imply the existence of an intergroup level within the research. In 2022, the total population of Tangerang Live application downloads was 213,646 individuals, whereas 400 people across 13 districts did not download the application (Table 3).

**Table 3:** Distribution of respondent samples [14].

No.	District	Respondents who downloaded Tangerang LIVE
1.	Karawaci	46
2.	Pinang	42
3.	Cipondoh	40
4.	Tangerang	39
5.	Cibodas	38
6.	Periuk	32
7.	Neglasari	30
8.	Larangan	27
9.	Ciledug	26
10.	Karang Tengah	24
11.	Jatiuwung	21
12.	Batu Ceper	19
13.	Benda	15
	**Total**	**400**

Observations were conducted on productive-age individuals who used the Live application in 13 districts. Apart from the users of Tangerang Live application, observations were also made regarding the State Civil Apparatus at the Tangerang city communication and information service, which serves as the manager of Tangerang Live application, as well as the technology infrastructure supporting this application.

### 3.3 Data processing

Descriptive analysis was used to analyze respondents' demographics, regarding gender, age, recent education, marital status, and occupation. This is conducted to determine the identity of the respondent, and represents the collection of data on each respondent's demographic using SEM (Structure Equation Modeling) software. Data processing is carried out for 1 month (May 2022). The outer model testing phase includes assessing the validity & reliability of indicators and constructs. The goodness of fit model testing stage involves evaluating the prediction power, the feasibility of the model, and the significance of the influence of exogenous variables on the endogenous (see Table 4).

**Table 4:** Characteristics of most respondents [14].

No	Description	Scale	Percentage
1	Age	between 15 and 21 Years Old	55%.
2	Gender	Female	51%
3	Final Education	Senior High School	72%
4	Marital Status	Unmarried	62%
5	Occupation	Employee	42%

## 4 Results

### 4.1 Respondent profile

There were several questions in the research questionnaire that measuring the objective condition of the respondents related to demographics profile, including age, gender, marital status, last education, and occupation. The description of the respondents represented by the five profile questions is as follows:

1. From the results, the age ranges from 15 to 21 years old (55%), indicate that the millennial age is very dependent on the application in facilitating their service of interest. For example, by utilizing the provided application options, changes to identity cards, such as applying for modification, can be implemented efficiently.
2. Many application users are females, as this gender uses the application for various services, such as accessing online market. The online market menu provides a choice of types of products, including vegetables, meat fruits, etc. Besides, there are also product promo notifications from every traditional and modern market in the city.
3. When viewed from the book entitled “Tangerang city in numbers for the year 2022" (Tangerang Municipality in Figures) published by the central statistics agency, it is stated that as many as 564,747 individuals of productive age from the age of 15 years onwards have completed high school education. This figure is 49.6% of the total productive age in Tangerang city, which is 1,141,720 people.
4. There are many Tangerang Live application services that are indeed needed by unmarried respondents, such as the plesiran choice menu. This feature provides information for tourist attractions in Tangerang City area, by meeting the respondents' need to explore and discover local destinations. Tangerang Live application offers transportation routes for various modes of transportation, facilitating access to the tourist attractions. It also provides valuable information regarding the historical background of these attractions, enhancing the overall experience for users.
5. Employees are synonymous with workers in the private sector who have their time consumed by work in the office, making it difficult to manage population administration documents effectively. Tangerang Live application has captured the interest of private employee due to its efficient in the management of population documents. One notable service is the neighborhood association introduction service, which offers various services such as assistance in obtaining certificates of death, widow/widower, and Taxpayer Identification Number.

### 4.2 Construct reliability and validity

According to Table 5, based on the validity and reliability analysis described above, it is known that all question items used in the research questionnaire have met the expected criteria. The validity criterion should exceed 0.300, whereas the reliability value should be surpassed 0.60.

**Table 5:** Reliability and validity.

	Cronbach's Alpha	rho_A	Composite Reliability	Average Variance Extracted (AVE)
Digital divide	0,844	0,849	0,906	0,762
Implementation Analysis Framework	0,914	0,915	0,939	0,795
Community need ICT based Smart City	0,847	0,851	0,897	0,686
Smart City Policy	0,763	0,763	0,864	0,679

### 4.3 Hypothesis testing

In this section, hypothesis testing will be carried out based on the results of the analysis using the partial least square method. The results of the analysis obtained showed that, of the five hypotheses put forward, all proved to be statistically significant. The results of more complete analysis and hypothesis testing can be seen in the next description.

**Fig. 2:**
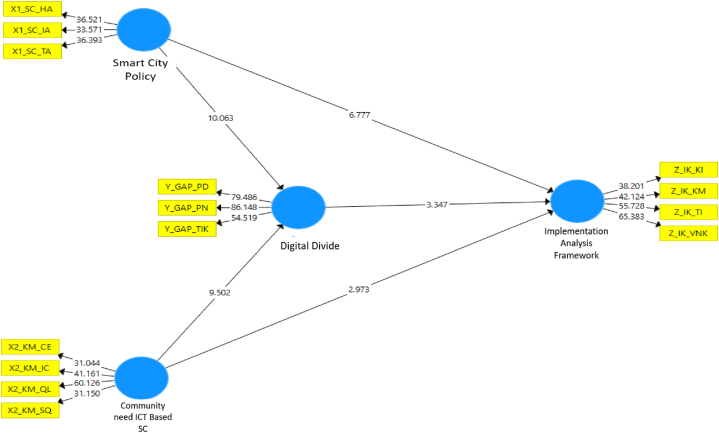
T-Value graph.

**Fig. 3:**
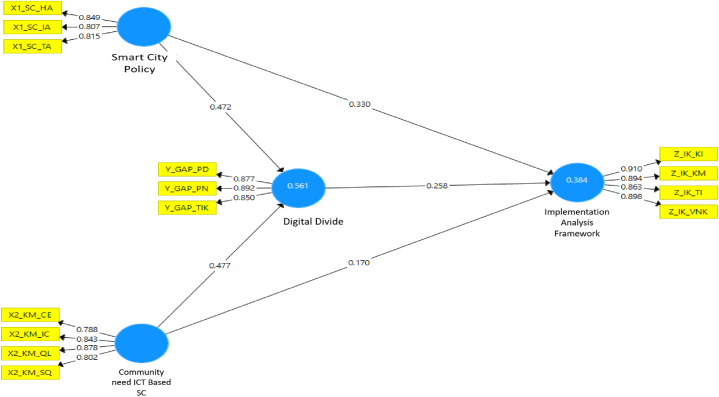
Path coefficient graph.

Fig. 2 indicates the significance value of the hypothesized relationship between variables. Relationships or paths that have an absolute value greater than 1.96 are declared statistically significant, whereas the smaller values are insignificant. Fig. 3 below indicates the weight or coefficient of relations between the variables obtained. The greater the value, the stronger the relationship formed between the two variables. It can be seen that the strongest relationship is formed between the community needs for digital divide (gap) with a value of 0.477 or 47.7%.

The results from Fig. 2, Fig. 3 above can be summarized into Table 6 as follows:

**Table 6:** Coefficient value and T-value.

Description	Coefficient	T-Value	Conclusion
Smart city policy - > Digital divide	0.472	10.06	Significant
Community needs - > Digital divide	0.477	9.50	Significant
Smart city policy - > Policy implementation	0.330	6.78	Significant
Community needs - > Policy implementation	0.170	2.97	Significant
Digital divide - > Policy implementation	0.258	3.34	Significant

Based on Table 6, the results of hypothesis testing show that the smart city policy has a positive significant influence on the digital divide with a loading of 0.472 or an influence of 0.472^2^, amounting 0.2227 (22.27%). The community needs have a positive significant impact on the digital divide with a loading of 0.477 or an influence of 0.477^2^ accounting for 0.2275 (22.75%). Smart city policy has a positive significant effect on policy implementation with a loading of 0.330 or an influence of 0.330^2^ amounting 0.1089 (10.89%). The community needs variable has a positive significant influence on policy implementation with a loading of 0.17 or an influence of 0.17 2 accounting for 0.0289 (2.89%). The digital divide has a positive impact on policy implementation with a loading of 0.258 or an influence of 0.258^2^ amounting 0.067 (6.7%).

## 5 Discussion

In the context of implementing smart city, the implementation of policy, has been reflected in the executive decree as stated in Tangerang Mayor Regulation Number 108 of 2018 concerning the Tangerang smart city masterplan 2017–2027. Mayor Regulation Number 108 shows the full support of the executive to build and develop Tangerang as a smart city, which will further drive the bureaucratic machinery or apparatus. In addition, the issuance of Mayor Regulation Number 108 can also be an indication of the support and seriousness of the Tangerang City regional government in terms of implementing smart city.

The existence of executive decisions as stated in Mayor Regulation Number 108 above is in line with the research that highlighted the importance of policy implementation at the executive level. Previous research [97] indicated that the execution of policy may be carried out starting with planning, technical direction, socialization, distribution of infrastructure, monitoring, and evaluation. This implies that the implementation of smart city and all its components, including the smart economy, environment, governance, living, mobility, and people, should be founded on a master plan and certain strategic planning. With a well-defined and focused policy, the responsibilities of each regional apparatus can be clearly outlined, enabling the mapping of essential resources, particularly for human resource development. Once all responsibilities and resources have been obtained, the process of developing smart city can commence, addressing and overcoming any remaining obstacles or challenges that may arise. The majority of smart city efforts are led by (local) governments and depend on the assistance of ICT to provide public services to the community, although they may vary from e-government initiatives in certain situations, there are many parallels between the two types of initiatives [98].

Some of the existing Tangerang Live application innovations include 1) Open API (Application Program Interface) policy including data standardization, integration, and public API development. 2) Development of a collaboration system with the Penta helix model that involves the local governments, universities/academics, private sector, society, and the media. 3) Integrated services, including the Tangerang Live and one-stop-shop concept for all services.

The local government through the communication and information Service implements Tangerang Live application as a digital platform for the third space of citizens consisting of 1) a digital society, where Tangerang Live application will facilitate interaction between the community, 2) The digital economy, bridging the interaction between the community and the business sector, to make the economy run in accordance with the government's expectations, and 3) Digital government, where interaction between the community and the government becomes easier through applications.

Tangerang Live application initiated by the government has several advantages, including 1) Effective, facilitating services in the Tangerang City area. 2) Innovative, Tangerang Live application will always be developed and have innovations to implement the smart city concept. 3) Easy to use, the Tangerang Live start page has a user-friendly and minimalist design, making it simple and easy to navigate. 4) Feature-rich, various features are found in Tangerang Live application. Smart city policy of Tangerang can affect the overall implementation of ICT policy by accelerating and increasing the use of communication technology in supporting the development of a smarter, more efficient, and sustainable Tangerang City.

The variety of complexities that exist in the city which tends to be unavoided will in turn increase pressure in the economy (poverty, unemployment), social (congestion, crime), and the environment (pollution, garbage, global warming). Smart city initiatives are employed to address the complexity of problems faced by modern cities. The implementation of smart city will have implications for the need to issue supporting policies and regulations, which is necessary in transforming a city into a smart city.

The policy has an influence on the digital divide since smart city requires access to good technology and infrastructure to operate effectively. However, not all residents have the same access to technology and the internet. When the smart city policy only focuses on developing technology and infrastructure, without paying attention to the gap among the residents, there will be a digital divide. This can result in inequalities in opportunities to access digital information and services, as well as gaps in the ability to benefit from technology and innovation.

To overcome the impact of the digital divide from smart city policy, the regional government should ensure that all citizens of the city have equal access to technology and the internet. One method to achieve this is by enhancing the accessibility of technology infrastructure, while offering training and education programs to individuals who have limited skills in using technology. By this method, the digital divide can be reduced and opportunities to leverage technology and innovation can be extended to all citizens of the city.

Policy implementation appears to be at a relatively good level. It is important to note that this analysis uses hypothetical numbers and considers only four finite factors. For a more comprehensive evaluation, more complete data and a detailed assessment of various aspects of policy implementation are needed, such as resource management, public participation, and policy impact evaluation. The digital divide can affect the implementation of policy designed with the assumption that all citizens have equal access to technology, and difficulties may arise when there is gap among residents within the city.

For example, when a smart city policy in Tangerang is designed to optimize online services (Tangerang Live application) and the use of technology in various aspects, but not all city residents have adequate internet access and technological devices, then the policy will not work effectively and may fail in its implementation. The impact of the digital divide can also affect the participation of the residents in the policy process. Residents who do not have adequate access to technology and internet may not be able to obtain information and engage in the public participation process conducted online. This can reduce the participation of citizens and the impact can reduce the effectiveness of the policy implemented.

To overcome the impact of the digital divide on policy implementation, the government should ensure that the policy implemented pays attention to the technology gap among residents. Governments can improve the accessibility of technology infrastructure, provide training, and education about technology to people who are less skilled in using technology. In this manner, policy implementation can run more effectively and public participation can be increased. Communities greatly benefit from ICT innovation, active participation in urban contexts, high-quality services, and an improved quality of life through the use of ICT. A significant positive change and development can be fostered by enhancing these aspects. To meet this need, investment in the development is required as well as implementation of innovative ICT solutions, attention to community participation, improving the quality of services, and ensuring equitable access.

Increasing needs for ICT can widen the digital divide as not all city residents have equal access to technology and the internet, resulting in technological gap among the citizens. This gap has an impact on the opportunity for the people to access information, utilize digital services, and benefit from innovation. With the development of ICT, the gap can further deepen the economic and social gap between residents. When the community's need for ICT is managed properly, this can affect the reduction of the digital divide in the city. The government should pay attention to the needs of the community for ICT and ensure that the technological infrastructure built cover all city residents. In addition, the administration should also provide training and education on ICT for people who are less skilled in using technology.

The implementation of policy is important to meet the needs of the people for ICT, which include 1) The development of adequate infrastructure can facilitate access. This includes the construction of extensive and quality internet networks, provision of cell phones, public Wi-Fi, hotspots, etc. 2) Training and development of human resources is needed to improve the technology skill of the resident's community through live training or online courses. 3) Regulations and policies that can help people access services and information online safely. This includes personal data protection, cybersecurity, and copyright. 4) Empowering micro, small, and medium enterprises (MSMEs) in the use of ICT can help them improve the quality of products and services and expand market reach. Implementation of this policy is expected to assist the people of Tangerang City in fulfilling their ICT needs, thereby enhancing their quality of life and welfare.

## 6 Conclusion

The Community Needs Variable has a positive significant impact on the Gap with a loading of 0.477 or an influence of 0.477^2^ accounting for 0.2275 (22.75%) of the relationship. This is the strongest relationship value, considering the constant changes and developments that the city undergoes, specifically in the rapid evolving field of ICT. Change often occurs in a very fast and unstoppable time, and in turn has an impact on the patterns and lifestyle of the people. The results and various research indicate that the digital divide in smart city context is evident in the work culture and the availability/support of sufficient ICT infrastructure. The digital divide arises because of a paradigm shift in work culture, from the old manual work culture to automation in the digital era (cashless, paperless, *etc*). To address this, policy promoting digital mindset and enhancing human resources capacity through various forms of training and education should be implemented, enabling individuals to effectively utilize ICT devices. To enhance support and availability of adequate ict infrastructure, one approach is to establish collaboration with external parties.

The community needs have a positive significant effect on Policy Implementation with a loading of 0.17 or an influence of 0.17 2, accounting for 0.0289 (2.89%) of the relationship. This is the weakest relationship value, where the complexity of the problem is increasing, ranging from the need to build green open space, decent housing, adequate infrastructure, support for business space (business), and the availability of recreational facilities. Additionally, there are challenges in formulating regulations that effectively support smart city initiatives.

One of the research limitation is the lack of indepth exploration regarding policy implementation strategies that are in accordance with the conditions of bureaucratic development in Tangerang City, specifically, and Indonesia in general. This include a need to investigate appropriate forms of work and support in the context of regional work tools specific to Tangerang. Another aspect that could have been explored is the influence of culture, as it plays a significant role in determining the applicability of successful strategies implemented in other cities and countries to the context of Indonesia, particularly Tangerang. Cultural considerations may necessitate modifications and adaptations to ensure the effectiveness of these strategies.

## 7 Implication

Based on the conclusions stated above, the following will outline the implications of research both theoretically and practically.

### 7.1 Theoretical implications

To measure the effect of policy related to the implementation of smart city in the future, the regional government should be able to prepare various regulations needed. The findings provide further support to the notion that leadership plays a crucial role in the successful implementation of smart city policy, highlighting its positive influence. Future research can consider the leadership factors needed for policy implementation, particularly focusing on the role of effective communication as a bridge between stakeholders in formulating regulations in accordance with the objective conditions encountered in the field. This will mitigate the digital divide that may arise in the implementation of smart city, both at the level of regional devices as service providers and the community as recipients of services. This is essential as the primary objective of smart city to provide better public services.

### 7.2 Practical implications

Based on the results, it is observed that policy implementation plays an important role in smart city development. Therefore, local governments and mayors as regional leaders can formulate the necessary regulations and demonstrate leadership capabilities in the context of implementing smart city. In an effort to reduce digital divide as an inevitable factor influencing the implementation of smart city, there is need for breakthroughs in terms of providing digital infrastructure, as well as building efforts that can encourage and also involve various stakeholders. When policy implementation is supported by proper guidance, the availability of adequate digital infrastructure support and the involvement of external elements can help address potential digital divide that may arise from smart city implementation.

## 8 Practical suggestions

1. In addition to the importance of policy implementation in supporting the realization of smart city, local governments should prioritize and encourage efforts to involve various stakeholders and communities through practical initiatives and collaborations. Efforts to promote external involvement are crucial to bridge the implementation gaps in the context of limited existing human resources (state civil apparatus). Furthermore, when involving communities or private parties, it is important to prioritize the implementation of directed and sustainable education and training programs. This approach aims to address the existing knowledge gap effectively and ensure adequate skill development.
2. For future research should consider the need for more in-depth exploration related to the forms of policy implementation that align with the actual conditions of bureaucratic development, specifically in Tangerang City and more broadly in Indonesia. This research should also examine the appropriate forms of work and support in the context of regional work tools. In this case, cultural considerations should also be taken into account, as what may have been successfully implemented in other countries may require modifications when applied in the context of Indonesia.

## Author contributions

Toddy Aditya; Conceptualization, research method, formal analysis, writing—original draft preparation, writing—review and editing.

Sinta Ningrum, Heru Nurasa. and Ira Irawati; project administration, supervision.

All authors have read and agreed to the published version of the manuscript.

## Funding

This research was funded by the Education Fund Management Institution of Lembaga Pengelola Dana Pendidikan (LPDP), (No: 20200421351518 for Toddy Aditya). The APC was funded by Universitas Padjadjaran.

## Institutional review board statement

Not applicable.

## Informed consent statement

Not applicable.

## Data availability statement

All the data are in the manuscript.

## Additional information

No additional information is available for this paper.

## Declaration of competing interest

The authors declare that they have no known competing financial interests or personal relationships that could have appeared to influence the work reported in this paper.
