# Supplementary material for: Community needs for the digital divide on the smart city policy
Source: Heliyon. 2023 Aug 4;9(8):e18932. doi: 10.1016/j.heliyon.2023.e18932 (PMC10425902; doi:10.1016/j.heliyon.2023.e18932)
Supplement: Multimedia component 1 [file mmc1.pdf]

UNIVERSITAS PADJADJARAN

DIRECTORATE OF RESEARCH AND COMMUNITY ENGAGEMENT

PROOFREADING CERTIFICATE

This document certifies that the manuscript listed below has been proofread for proper English language, grammar, punctuation, spelling by one or more of the qualified proofreaders.

Manuscript title:

Community Needs for the Digital Divide on the Smart City Policy

Author(s):

Toddy Aditya, Sinta Ningrum, Heru Nurasa, Ira Irawati

Date issued:

June 14th, 2023

Certificate Verification Key:

DRPM/PR/1112.06/2023

Neither the research content nor the authors' intentions were altered in any way during the proofreading process. Documents receiving this certification should be English-ready for publication, however, the author has the ability to accept or reject our suggestions and changes. Should you have any questions or concerns about this proofread document, please contact proofreading clinic at riset@unpad.ac.id

Proofreading Clinic | Copyright © 2017 DRPMI UNPAD. All rights reserved
